# The retail food environment following a mandatory healthy checkout policy: A natural experimental evaluation

**DOI:** 10.1371/journal.pmed.1004814

**Published:** 2026-09-16

**Authors:** Yuru Huang, Ethan C. Wolf, Andrea M. Gil, Kris Jayme G. Matas, Lisa M. Powell, Rebecca M. Schermbeck, Jennifer Falbe

**Affiliations:** 1 Human Development and Family Studies Program, Department of Human Ecology, University of California, Davis, California, United States of America; 2 Department of Preventive Medicine, University of Tennessee Health Science Center, Memphis, Tennessee, United States of America; 3 Bursky School of Public Health, Washington University in St. Louis, Missouri, United States of America; 4 Public Health Nutrition Program, Community Health Sciences, UC Berkeley School of Public Health, Berkeley, California, United States of America; 5 Graduate Group in Nutritional Biology, Department of Nutrition, University of California, Davis, California, United States of America; 6 Institute of Global Nutrition, University of California, Davis, California, United States of America; 7 Health Policy and Administration, School of Public Health, University of Illinois Chicago, Chicago, Illinois, United States of America; 8 Institute for Health Research and Policy, School of Public Health, University of Illinois Chicago, Chicago, Illinois, United States of America; Waymark, UNITED STATES OF AMERICA

## Abstract

**Background:**

In January 2024, Perris, California, became the third jurisdiction worldwide to implement a mandatory healthy checkout policy, after Berkeley, California, and England. Healthy checkout policies set nutrition standards for products displayed at checkout, with the potential to improve the healthfulness of the retail food environment and ultimately dietary intake. Policy scope varies across jurisdications, with Berkeley’s and England’s applying to the entire checkout, while Perris’ applies to products situated within a 6 ft radius of the register. The real-world impact of these policies on the retail food environment is not well established.

**Methods and findings:**

We leveraged a natural experiment, collecting data in June 2023 (baseline) and June 2024 (follow-up; 6 months after implementation) from the 13 Perris stores subject to its healthy checkout ordinance (HCO) and 51 matched stores in three comparison cities without the policy. Using 65,271 product facings (i.e., each product that faces consumers from which they can select), we conducted a difference-in-differences analysis to assess the extent to which: (1) checkout product facings met HCO standards ≤6 ft of registers (i.e., were HCO-compliant [the policy applies only in this zone]), >6 ft of registers, and across the entire checkout; and (2) checkout food and beverage facings fell into compliant and noncompliant categories ≤6 ft. Among all product facings ≤6 ft of registers, compliance with the Perris HCO standards increased from 50% to 67%, a 37% increase relative to comparison cities (PR = 1.37; 95% CI [1.12,1.68]; *p = 0.002*). Increases in the percentage of facings meeting HCO standards were not significant >6 ft of registers and were smaller across the entire checkout (PR = 1.31; 95% CI [1.06,1.61]; *p = 0.011*). Among food and beverage facings ≤6 ft of registers, compliance increased 52% relative to comparison cities (PR = 1.52; 95% CI [1.11, 2.07]; *p = 0.009*)*,* from 36% to 55%; there was a 141% relative increase in compliant beverages (from 6% to 18%, PR = 2.41; 95% CI [1.40,4.14]; *p = 0.001*) and a 200% relative increase in 100% fruit juice (from 0.7% to 1.7%, PR = 3.00; 95% CI [2.04,4.41]; *p < 0.001*)*.* However, there were no significant reductions in the percentage of food and beverage facings ≤6 ft that belonged in any specific noncompliant categories: candy, sugar-sweetened beverages, salty snacks, or other sweets. A limitation of this analysis is that it assumed but could not verify parallel trends; if this assumption was not met, the results could be biased.

**Conclusions:**

Mandatory HCOs improve checkout food environments, but their impact may in part depend on the policy’s design. The Perris HCO significantly improved the presence of healthier foods and beverages ≤6 ft of registers but not for the rest of the checkout area. Jurisdictions considering HCOs should carefully define policy scope and nutrition standards to maximize impact.

## Author summary

**Why was this study done?:**

- Most checkout foods and beverages are unhealthy (e.g., candy, sugary drinks), which may encourage unhealthy impulse purchases.
- In January 2024, Perris, California, became the third jurisdiction worldwide to implement a mandatory healthy checkout policy, targeting products within 6 ft of registers.
- Few jurisdictions have implemented mandatory healthy checkout policies, and evidence on their real-world impact is limited.

**What did the researchers do and find?:**

- We evaluated changes in the retail checkout food environment in 13 Perris stores and 51 matched comparison stores before and six months after implementation, using a difference-in-differences design.
- Within 6 ft of registers, compliance increased from 50% to 67% (37% relative increase versus comparison cities), with increases in compliant beverages and 100% fruit juice (with no added caloric sweeteners).
- No significant reductions were found in specific noncompliant categories (candy, sugar-sweetened beverages, salty snacks, other sweets), and improvements beyond 6 ft were not statistically significant.

**What do these findings mean?:**

- Mandatory HCOs improve checkout food environments, but the extent of improvement depends on policy design.
- Jurisdictions considering HCOs should apply broader checkout definitions (e.g., the entire checkout area rather than just 6 ft from registers) and more comprehensive nutrition standards to maximize policy impact.
- A limitation of the study is that it assumed without the policy, the checkout food environment in Perris would have changed similarly to that in the comparison cities, which could introduce bias if this was not the case.

## Abstract 3

In this natural experiment study, Huang and colleagues show that the mandatory healthy checkout policy in Perris, California, is associated with a notable increase in healthier food and beverage products displayed near cash registers, where the policy applies. The findings suggest that checkout policies can help improve the healthfulness of retail food environments, with effects appearing to depend on how the policy is designed and implemented.

## Introduction

In the United States (U.S.) and globally, store checkouts are often stocked with unhealthy foods and beverages, such as candy, salty snacks, and sugary drinks [1,2]. This strategic placement is paid for by manufacturers through marketing agreements and is known to encourage impulse purchases while customers wait in line to pay [3–5]. With 36% of U.S. adults purchasing foods or beverages found at checkout [6], and greater exposure to unhealthy checkouts in neighborhoods with lower socioeconomic status and higher Black and Hispanic composition [7], these marketing practices may harm population dietary quality and equity. Because the retail food environment influences consumer purchases, and due to the salience of the checkout lane, policies that regulate product placement in the checkout lane have emerged as a novel public health tool [6,8–10].

In January 2024, Perris, California (CA), implemented a mandatory healthy checkout policy [11], becoming the second jurisdiction in the U.S. (after Berkeley, CA [12]) and the third worldwide (after England [13]) to do so. At checkouts, Perris’ healthy checkout ordinance (HCO) permits only foods and beverages meeting the HCO’s nutrition standards, such as water without caloric sweeteners, low-calorie beverages, fruits, vegetables, nuts, seeds, and legumes [11] (see Methods for full standards; Table 1). The policy applies to all products within a 6 ft radius of any register in large commercial establishments (>2,500 ft^2^) that sell groceries in the city. The stated rationale for the HCO is to “support families by offering them healthy food and beverage items at checkout aisles and the choice to avoid high-calorie, low-nutrient food when they do their grocery shopping” [11].

**Table 1:** Perris Healthy Checkout Ordinance standards (“healthy food options”).

Section	Verbatim Policy Text (Ord. No. 1433, § 3, 9-12-2023)
**(a) Beverages: A commercial establishment with groceries that sells beverage items at the checkout aisle shall make the default beverage options the following:**	
(1)	Water, including carbonated water with no added caloric sweeteners;
(2)	Coffee or tea with no added caloric sweeteners (permissible condiments include sugar, sugar substitutes, milk, and creamer products);
(3)	Fat-free or one percent low-fat dairy milk or calcium- and vitamin D-fortified soymilk with fewer than 200 calories per container;
(4)	One hundred percent fruit juice or fruit juice combined with water or carbonated water, with no added caloric sweeteners, in a size no greater than 20 fluid ounces;
(5)	One hundred percent vegetable juice with no added caloric sweeteners, no more than 200 mg of sodium per container, and in a size no greater than 20 fluid ounces; or
(6)	Low-calorie beverages that have no more than 40 calories per container.
**(b) Foods: A commercial establishment with groceries that sells food items at the checkout aisle shall make the default food items, per package, the following:**	
(1)	No more than 200 calories;
(2)	No more than 35 percent of calories (or 10 g) from total sugars;
(3)	No more than 200 mg of sodium; and
(4)	Meet at least one of the following standards or have the first ingredient on the ingredients list be:
(4a)	Sugar-free chewing gum or mint;
(4b)	Fruits or vegetables; or
(4c)	Nuts, seeds, or legumes; or
(4d)	Whole grains; or
(4e)	Low-fat or fat-free dairy.
*Exemption*	The requirements of subsections (1) through (3) of this section shall not apply to fruits, vegetables, nuts, seeds, and legumes.

Because few jurisdictions have implemented mandatory healthy checkout policies [12,13], evidence of their real-world impact on the retail food environment remains limited. While voluntary policies and experimental studies show that healthy checkouts can improve the healthfulness of purchases [14–18], evidence from mandatory real-world policies is needed to understand how retailers respond in practice. To date, the only two studies evaluating such policies at the product level have examined Berkeley’s HCO [19,20]. These studies found that 1 year after implementation, compliance with Berkeley’s HCO increased from 53% to 83%, and the presence of sugar-sweetened beverages (SSBs), candy, and other sweets significantly decreased [19], which corresponded to a 70% reduction in added sugars per serving for foods and beverages at checkout relative to comparison cities [20]. Additional evidence from Nottinghamshire, England, found that after England’s policy restricting the placement of high saturated fat, salt, or sugar (HFSS) products in prime locations, exposure to any restricted products in regulated locations in supermarkets fell from 39% to 14% [21].

Unlike England’s policy, Berkeley’s and Perris’ HCOs apply only to checkout areas. There are, however, important differences in HCO standards and scope between Berkeley and Perris that may affect how stores respond to such policies [11,12]: (1) Berkeley prohibits low-calorie SSBs and artificially sweetened beverages, while Perris has full allowance; (2) Berkeley sets *per-serving* nutrient limits for all foods, which aligns with U.S. nutrient labeling, whereas Perris sets *per-container* limits for calories and nutrients while exempting fruits, vegetables, nuts, seeds, and legumes from these limits; (3) Berkeley’s HCO covers all checkout areas, while Perris’ HCO applies to only ≤6 ft of registers, excluding some endcaps (where beverages are stocked) and parts of longer checkout lanes; and (4) types of stores subject to the ordinance differ (e.g., drugstores are subject in Berkeley but not Perris). Understanding how policy differences may influence compliance is a key first step in guiding the design of future healthy checkout policies, which are being adopted or actively considered internationally and in multiple U.S. jurisdictions [10,22–24].

This study examined whether, and to what extent, the healthfulness of products available at checkout changed six months after the policy took effect (one year after the originally scheduled date, which was delayed due to industry pushback [25]). We evaluated changes in the entire checkout food environment as well as ≤6 ft of registers. Our primary hypothesis was that the percentage of product facings meeting HCO standards within the policy zone would increase in Perris relative to comparison cities after policy implementation.

## Methods

### Study design

We leveraged a natural experiment to evaluate the Perris HCO by comparing changes in the healthfulness of product facings at store checkouts in Perris with those in three comparison cities where the policy was not implemented. Data on products available at store checkouts were collected in June 2023 (baseline; about one month before the policy’s originally scheduled start date and six months before actual policy implementation) and again in June 2024 (follow-up; about six months after the actual policy implementation). As shown in S1 Table, data were collected from a census of 13 stores in Perris that are subject to the ordinance, and 51 stores in Moreno Valley, Rialto, and Riverside, CA (“comparison cities”) that were open at baseline and follow-up.

The comparison cities were selected for their similarity to Perris in retail environments (i.e., chains available), income, education levels, and ethnic composition (see S2 Table). These sociodemographic characteristics are well-established correlates of the retail food environment [26–28], which strengthens the assumption of parallel trends between intervention and comparison cities. The Perris sample included six chain supermarkets, four independent grocery stores, two dollar stores, and one mass merchandiser. Comparison stores were selected using stratified random sampling from all eligible food retail stores within comparison cities. Stores were matched by chain where possible (7 of 9 chain stores in Perris had direct matches in comparison cities) and by store type otherwise. Within each stratum, stores were randomly selected a priori using a random number generator. We aimed to include at least three comparison stores per intervention store and to represent each store type across comparison cities; additional backup stores were initially sampled to account for potential closures. This study is reported as per the Strengthening the Reporting of Observational Studies in Epidemiology (STROBE) guideline (S1 Checklist).

### Data collection, entry, and validation

#### Data collection.

Trained data collectors visited each store, photographed products at checkouts, and assessed the characteristics of each product facing using a reliable photo-based protocol, the Store CheckOUt (SCOUT) tool [29]. If a store had more than three checkouts, random sampling of three checkouts was conducted a priori using a random number generator. Data collectors were provided with a list of the three checkouts and a randomly generated sequential list of alternative checkouts to assess in case a sampled checkout was inaccessible. If a store had a self-checkout area, one self-checkout lane was included. The same checkouts were assessed pre- and post-policy. If a lane was not accessible at follow-up, it was replaced with the next available lane of a corresponding even or odd number when possible. For example, if checkout lane 5 was assessed at baseline but unavailable at follow-up, another odd-numbered lane (e.g., 1, 3, 7, or 9) was selected.

#### Checkout area.

This study assessed the entire checkout area, which included four distinct types of locations: main checkout lanes and registers, checkout endcaps (displays at the end of checkout lanes), snaking sections (single winding lanes that typically lead to multiple registers), and standalone displays located within 1 ft of an endcap or lane.

#### Product facing data entry.

Once all images were collected, trained data coders entered information for each product facing, including brand name, flavor, size, and other relevant attributes (e.g., product category). Product facings were defined as each of the individual products that face consumers from which a product can be selected, excluding those stacked behind. Identical products placed side by side were each counted as separate facings.

#### Distance from registers.

We classified product facings as ≤6 ft or >6 ft from registers using a structured distance estimation protocol (S1 File). Two stores were directly measured on-site using the iPhone measuring app to calibrate photographic estimation for the same retail chains. Distance from registers at the remaining stores was determined by carefully referencing the photographs collected. The center point for establishing radii was defined as where a customer and cashier interact to perform a transaction, such as a card reader, scanner, or touchscreen. Zone boundaries were informed by the dimensions of flooring tiles, products, and physical structures using strategies such as point-perspective principles, digital-to-physical scaling, geometric mapping, and grid-based diagrams. These procedures ensured consistent classification across study periods, as the ≤6 ft requirement was introduced after baseline assessments, and distance from registers was therefore not measured at baseline.

#### Nutrient data entry.

Nutrition data for each product were obtained from manufacturers’ websites, or, when not available, from retailer websites and publicly available databases [1]. In addition to calories, total fat, saturated fat, sodium, fiber, total sugar, and added sugar, we collected serving size, serving size unit, servings per package, and ingredient information for each unique product. These data were then used to determine whether a product met HCO nutrient standards. If the exact product size or flavor was unavailable, we used the same product in another size or flavor and proportionally scaled nutrient values to calculate nutrients per container. For products for which nutrient data were not available and the food group could be ascertained from photos with high confidence (e.g., spring water, pumpkin seeds in the shell, gummy candy), compliance was determined based on food-group categories for which classification did not require nutrient data (e.g., water, seeds, candy).

A total of 68,978 product facings from 64 stores were captured (mean = 1,078 facings per store). Of these, 3,707 (5.4%) could not be assessed for compliance due to obstructions in photographs, missing nutritional information, or the product being out of stock at the time of data collection, and were excluded from analyses. The final analytic sample comprised 65,271 product facings, of which 50,553 (77%) were foods and beverages. Overall, 57% (36,915/65,271) of all facings were located ≤6 ft of registers.

### Outcome measures

The main study outcomes were: (1) whether a product facing met HCO standards, and (2) for food and beverage facings, its food or beverage category. We assessed the overall percentage of product facings that met HCO standards among those ≤6 ft of registers, those >6 ft of registers, and those across the entire checkout. Herein, “compliant” and “compliance” refer only to product facings ≤6 ft of registers, the policy’s applicable zone, that met the HCO standards. Nonfood and nonbeverage facings were deemed as meeting the HCO standards (i.e., were “compliant” if ≤6 ft). The primary nutrient standards of interest were those in the Perris HCO, but we also examined whether products met the Berkeley HCO standards for comparison.

#### Perris’ HCO standards: Food and beverage categories.

Food and beverage categories included healthier categories (meeting Perris’ HCO standards; see Table 1) or less healthy (not meeting the HCO standards) categories [11]. Foods or beverages were classified as not meeting HCO standards if they were a whole grain or low-fat/fat-free dairy product exceeding any applicable nutrient limits or if they were not included in a permissible food or beverage category. These categories not meeting HCO standards included candy, SSBs containing >40 kcal per container, salty snacks (e.g., chips, jerky, pretzels, crackers), other sweets (e.g., cookies, baked goods, cake/cupcake), and other foods (e.g., sauces, condiments, white bread). In our product classifications, all sugar-free gums/mints were assumed to meet standards, even when sold in large packages that could hypothetically exceed calorie limits, due to these products typically having imprecise calorie labeling (e.g., < 5 kcal).

These permissible categories differed somewhat from conventional food categories. For example, a candy with coconut as the first ingredient would be classified as “candy” conventionally, but as “fruit/vegetable” according to the ordinance, since its first ingredient is a fruit. As such, we also defined a stricter “fruit/vegetable” compliance category, limited to conventional categories of preserved, dried, fresh, and prepared vegetables and fruits.

#### Meeting Berkeley HCO standards.

Because the only existing study examining the food environment following implementation of a mandatory healthy checkout policy in the U.S. was conducted in Berkeley [19], we also assessed whether products met the Berkeley HCO standards to enable direct comparisons. Facings in Perris were coded as meeting the Berkeley HCO standards if they were unsweetened beverages (not sweetened with sugar or non-sugar sweeteners) or foods that contain ≤5 grams added sugars and ≤200 mg sodium in one of the following categories: sugar-free gum/mints, fruit, vegetables, nuts, seeds, legumes, yogurt or cheese, and whole grains [12]. Nonfood or nonbeverage products were also deemed to meet the standards.

### Statistical analysis

We first examined the total number of facings before and after the policy, in Perris and comparison cities. For the main outcomes, we used difference-in-differences (DiD) Poisson regression models with store-clustered robust standard errors to estimate whether changes from pre- to post-implementation in Perris differed from those in the comparison cities. Specifically, we looked at changes in (1) the percentage of all product facings that met the HCO standards and (2) the percentage of all food and beverage facings that belonged to each food and beverage category. The unit of analysis was product facings, and the DiD model was:


$$\begin{array}{c} \text{log}(E[Y_{it}])\;=\;\beta_{\text{0}}\;+\;\beta_{\text{1}}{\text{Intervention}}_{i}\;+\;\beta_{\text{2}}{\text{Post}}_{t}\;+\;\beta_{\text{3}}\;({\text{Intervention}}_{i}\times \;{\text{Post}}_{t}) \end{array}$$

where *Y*_*it*_ is the binary outcome for product at site *i* (Intervention: 1 = Perris intervention site, 0 = comparison cities) during time period *t* (Post: 0 = Baseline, 1 = Follow-up). Model outcomes were reported as probability ratios (PRs), which are the exponentiated interaction terms (*β*_3_), and marginal means were calculated to estimate the percentage of products before and after the implementation in intervention and comparison cities. Some food and beverage categories had too few product facings (e.g., no compliant vegetable juice), for which no separate DiD Poisson models were run.

We additionally stratified the analysis by store type (chain supermarket, mass merchandiser, dollar store, and independent grocery) and by checkout location (standalone display, endcap of checkout, snaking section, and main checkout). For analysis by store type, we focused on product facings ≤6 ft of registers to assess compliance. For analysis by checkout location, we included product facings at all distances, as most standalone displays, endcaps, and snaking sections were located outside the 6 ft radius.

Additional analyses included: (1) adjusting for store type to account for sample imbalance and assess its impact on compliance among all facings and among food and beverage facings (S1 Fig); and (2) applying Berkeley HCO standards to compare the percentages of all facings and food and beverage facings meeting Perris versus Berkeley HCO standards (S2 Fig).

## Results

### Absolute number of product facings

**Fig 1:**
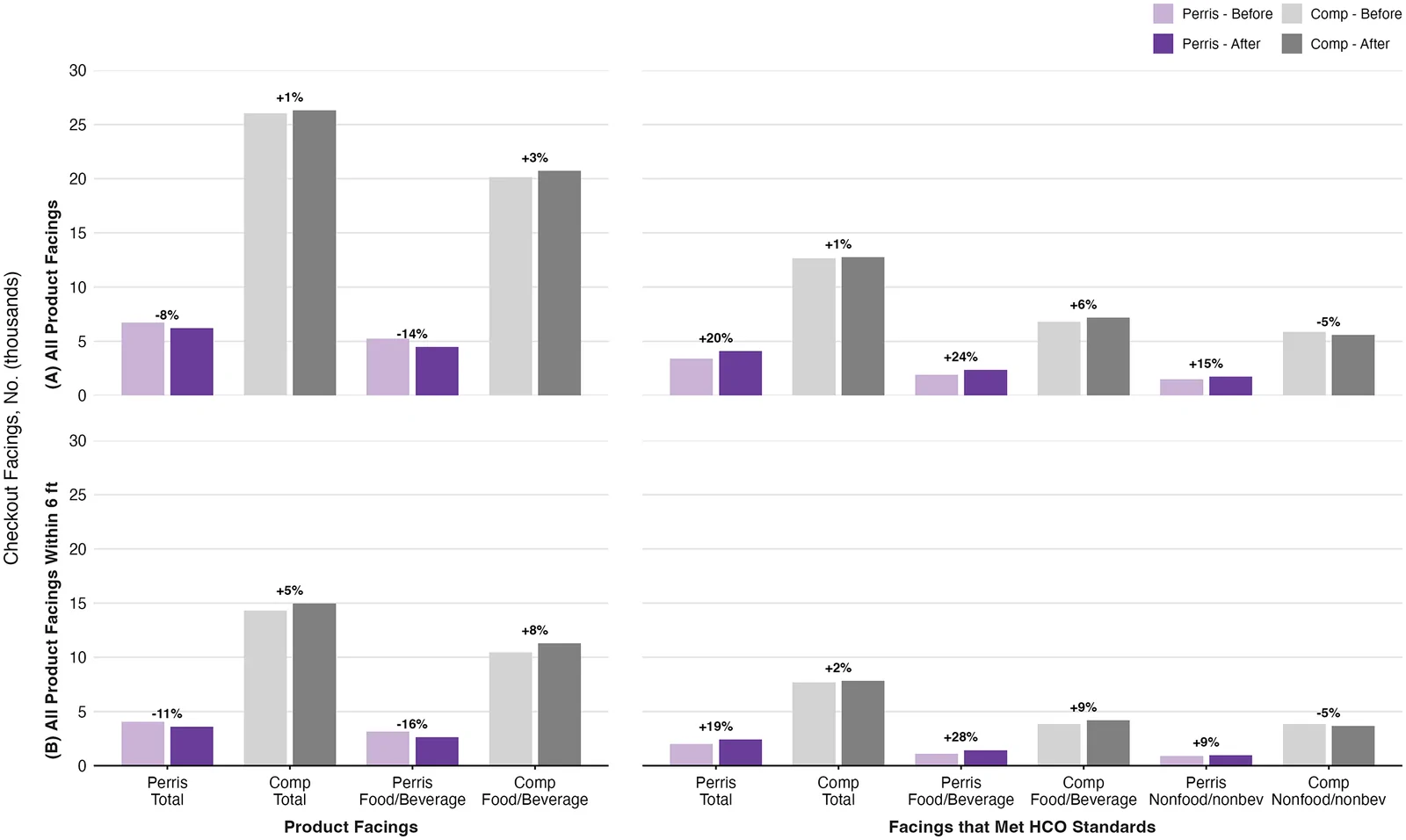
Total number of product facings and facings that met healthy checkout ordinance (HCO) standards before and six months after implementation of the policy in Perris and comparison cities, for all product facings (panel A) and facings within 6 ft (policy scope; panel B). **Abbreviations**: nonfood/nonbev, nonfood or nonbeverage; comp, comparison cities.

Fig 1 shows the counts of all product facings, food and beverage facings, as well as facings that met the HCO standards at checkout before and 6 months after the implementation of the HCO in Perris and comparison cities. The total product facing count at checkout decreased in Perris (−8%) and increased minimally in comparison cities (+1%). Within 6 ft of registers, the product facing count decreased substantially in Perris but increased in comparison cities, particularly for food and beverage facings (e.g., −16% in Perris versus +8% in comparison cities). Despite the decrease in total facings at checkout, the number of compliant facings in Perris increased, with a 19% (versus 2% in comparison cities) increase overall, 28% (versus 9%) increase for compliant food/beverage facings, and 9% (versus −5%) increase for compliant nonfood/nonbeverage facings. Similar increases in the number of facings that met the HCO standards were observed across the entire checkout area. S3 Fig presents these facing counts per store.

### Compliance among all facings within the policy zone (≤6 ft of registers)

**Fig 2:**
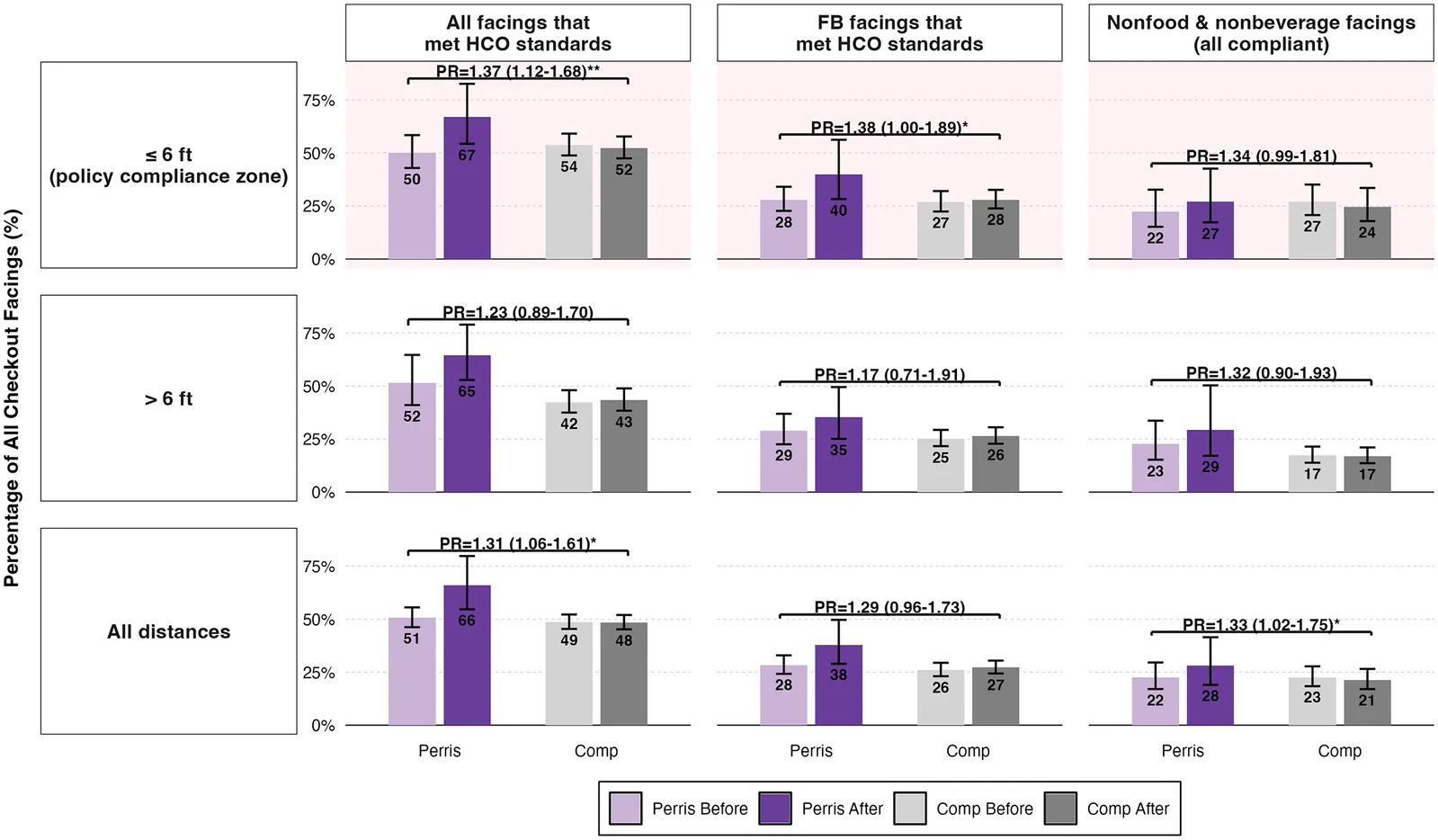
Percentage of all checkout facings that met HCO standards before and 6 months after implementation in Perris and comparison cities, shown for: (1) ≤6 ft (compliance, top panel, highlighted), (2) >6 ft (middle panel), and (3) all distances (bottom panel). All nonfood and nonbeverage facings met HCO standards. Abbreviations: PR, probability ratio; comp, comparison cities. ** *p* < 0.01, * *p* < 0.05. Denominators for each outcome were the total number of *p*roduct facings for the corresponding distance category. P values are from two-sided Wald tests of the difference-in-differences estimates, with standard errors clustered by store.

Fig 2 shows the percentage of all checkout facings that met the Perris HCO standards before and 6 months after implementation in Perris and comparison cities, along with the PR comparing them, for ≤6 ft (policy zone), > 6 ft, and the entire checkout (i.e., all distances combined). Within the policy zone, the percentage of all facings that were compliant with Perris’ HCO increased from 50% to 67% in Perris, a 37% (PR = 1.37; 95% CI [1.12,1.68]; *p = 0.002*) increase relative to the comparison cities, where the percentage remained stable (from 54% to 52%). This increase in compliance among all facings was driven by both increases in compliant food and beverage facings and increases in nonfood and nonbeverage facings, all of which were compliant. Specifically, the percentage of all facings that were compliant food and beverage facings increased from 28% to 40% in Perris, a 38% relative increase (PR = 1.38; 95% CI [1.00,1.89]; *p = 0.047*), while the percentage of all facings that were nonfood and nonbeverage facings showed a nonsignificant increase (PR = 1.34; 95% CI [0.99,1.81]; *p = 0.055*). These results were similar after adjusting for store type (S1 Fig). Noncompliant facings still constituted 33% of all product facings in the policy zone post-implementation.

### Percentage of facings meeting HCO standards across the entire checkout

When considering the entire checkout (i.e., all distances), some results were attenuated (Fig 2): There was a 31% increase in the percentage of all facings meeting HCO standards (PR = 1.31; 95% CI [1.06,1.61]; *p = 0.011*) from 51% to 66% and a nonsignificant change in the percentage of all facings that were foods and beverages meeting HCO standards (PR = 1.29; 95% CI [0.96,1.73]; *p = 0.097*) in Perris relative to comparison cities. Across the entire checkout, there was a 33% increase in the percentage of all facings that were nonfood and nonbeverage facings (PR = 1.33; 95% CI [1.02,1.75]; *p = 0.038*) in Perris relative to comparison cities (similar to ≤6 ft). For product facings >6 ft from registers (outside the policy zone) but still within the checkout area, increases in the percentage of facings that met HCO standards were also smaller and not statistically significant.

In analyses applying Berkeley’s HCO standards (S2 Fig), the absolute percentage of products that met Berkeley’s standards after implementation was lower (54% versus 67%), while the relative increase among all facings was similar to that observed using the Perris HCO standards (36% versus 31%; PR = 1.36; 95% CI [1.10,1.68]; *p* = 0.004).

### Percentage of food and beverage facings that were compliant within the policy zone (≤6 ft of registers)

**Fig 3:**
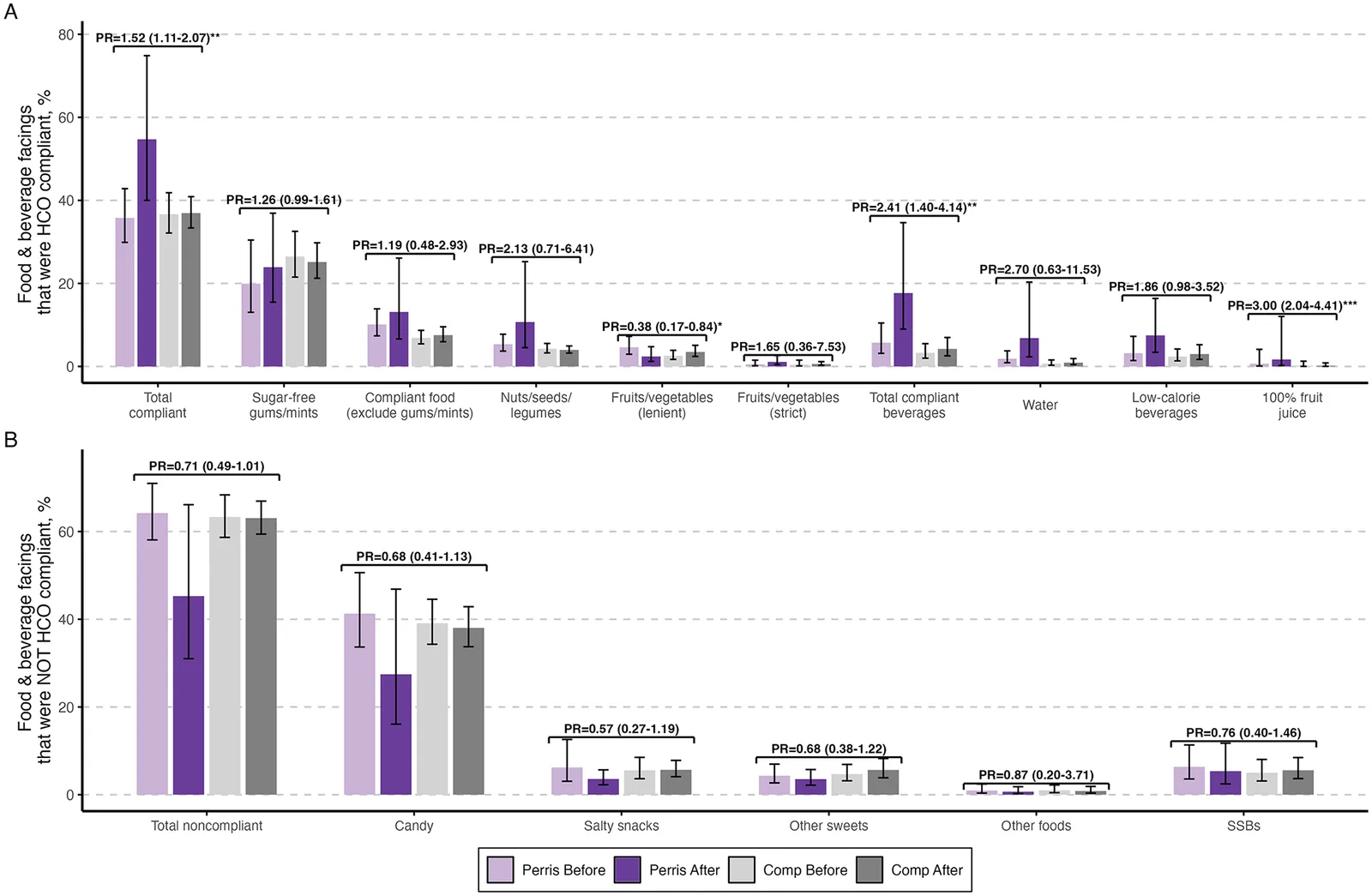
Percentage of food and beverage facings by healthy checkout ordinance (HCO) compliance category before and after implementation in Perris and comparison cities. Abbreviations: PR, probability ratio; comp, comparison cities; SSB, sugar-sweetened beverages. *** *p* < 0.001, ** *p* < 0.01, * *p* < 0.05. Denominators for each outcome were the total number of food and beverage product facings within 6 ft of registers. For panel **A**, total compliant = compliant foods (exclude gums/mints) + sugar-free gums/mints + total compliant beverages. Salty snacks include chips, jerky, pretzels, and crackers; other sweets include cookies, baked goods, and cake/cupcakes; other foods include sauces, condiments, and bread. A lenient definition for fruit and vegetable considers only the first ingredient regardless of conventional category (e.g., candy), whereas a strict definition is limited to preserved, dried, fresh, and prepared vegetables and fruits. Probability ratios for compliant and noncompliant categories are not exact inverses due to the log link in Poisson regression; their significance levels may therefore differ. P values are from separate two-sided Wald tests of the difference-in-differences estimate for each product category, with standard errors clustered by store.

Fig 3 shows results ≤6 ft of registers by compliance category when only food and beverage facings were considered. The percentage of food and beverage facings that were compliant with Perris’ HCO increased by 52% (PR = 1.52; 95% CI [1.11,2.07]; *p = 0.009*) in Perris (from 36% to 55%), relative to the comparison cities (no change; 37% to 37%). These results were similar after adjusting for store type (S1 Fig). In analyses applying Berkeley’s HCO standards (S2 Fig), this change was similar (PR = 1.52; 95% CI [1.09,2.11]; *p = 0.014*).

When examining category-specific changes in the percentage of food and beverage facings that were compliant with Perris’ HCO standards, there was a 141% increase in compliant beverages (from 6% to 18%; PR = 2.41; 95% CI [1.40,4.14]; *p = 0.001*); a 200% increase in 100% fruit juice with no added caloric sweeteners, which is a subset of compliant beverages (from 0.7% to 1.7%; PR = 3.00; 95% CI [2.04,4.41]; *p* < 0.001); and a 62% decrease in fruits/vegetables [lenient definition] (from 4.6% to 2.4%; PR = 0.38; 95% CI [0.17, 0.84]; *p = 0.017*) in Perris relative to the comparison cities (Fig 3). However, the decrease in fruits/vegetables under the lenient definition was mostly driven by chips and candies whose first ingredient was a fruit or vegetable; under the strict definition, fruits/vegetables increased, although not significantly (from 0.5% to 1.1%; PR = 1.65; 95% CI [0.36, 7.53]; *p* = 0.521). Other individual category changes (e.g., 113% increase in nuts/seeds/legumes, 170% increase in water, 86% increase in low-calorie beverages, and 26% increase in sugar-free gums/mints) were not statistically significant in Perris relative to the comparison cities.

The percentage of food and beverage checkout facings in Perris that were noncompliant with Perris’ HCO standards decreased marginally by 29% (from 64% to 45%; PR = 0.71; 95% CI [0.49, 1.01]; *p = 0.059*) relative to comparison cities (no change; 63% to 63%; Fig 3). Decreases in the percentage of all food and beverage facings that fell into specific noncompliant categories (e.g., 32% decrease in candy, 43% decrease in salty snacks, 32% decrease in other sweets, and 24% decrease in SSBs) were not statistically significant in Perris relative to comparison cities.

### Changes by checkout location

**Fig 4:**
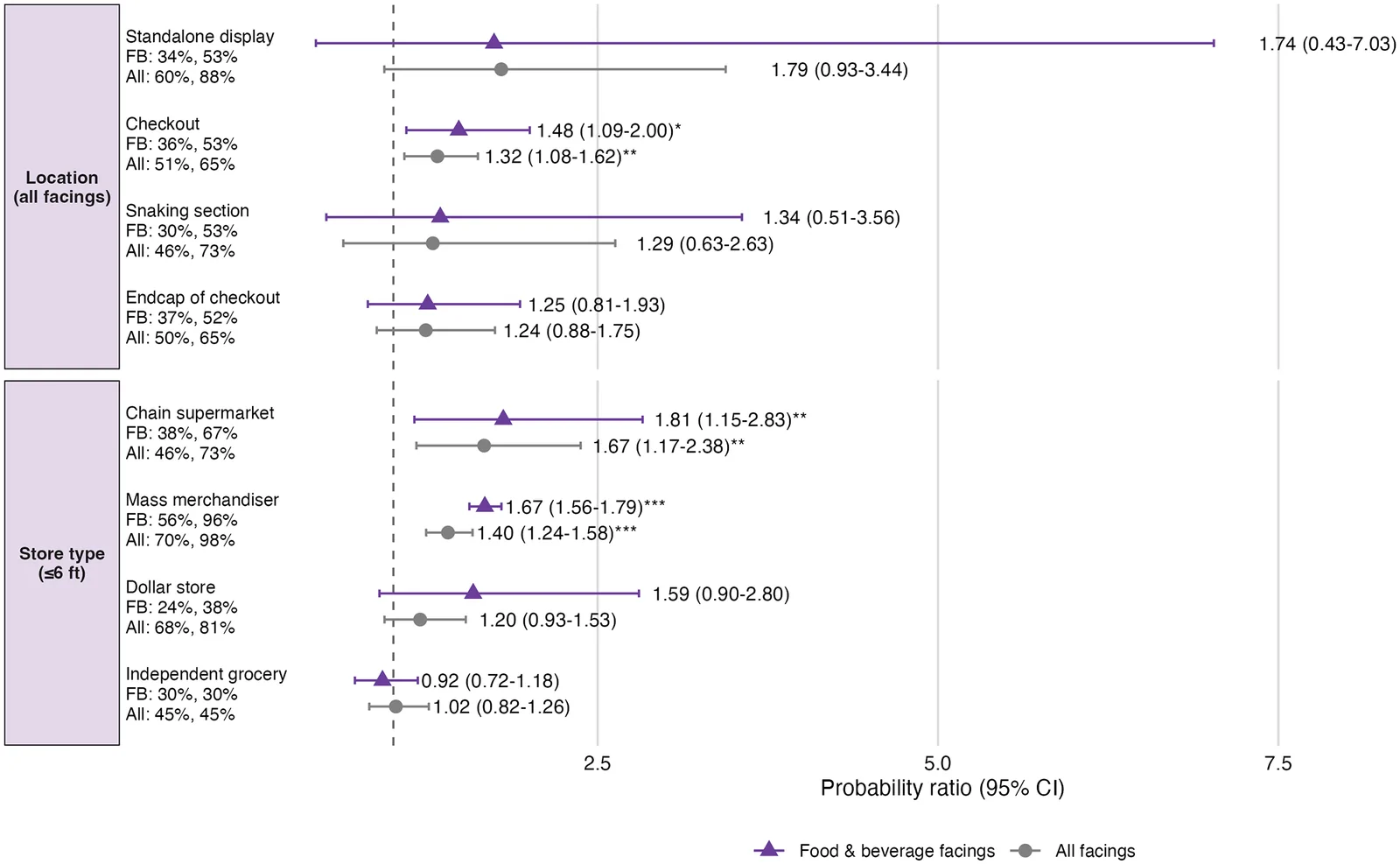
Probability ratios for the percentage of checkout facings meeting Perris’ healthy checkout ordinance (HCO) standards in Perris relative to comparison stores, by location within the checkout and store type. Estimates are shown by checkout location using facings at all distances (top panel) and by store type using facings within ≤6 ft of registers (bottom panel). Each panel includes estimates for all product facings and food and beverage facings. Abbreviations: FB, food and beverage facings; All, all product facings;***p < 0.001, **p < 0.01, *p < 0.05. *P* values are from separate two-sided Wald tests within each checkout location or store-type group, with standard errors clustered by store.

Fig 4 shows the PRs for the percentage of all facings and food and beverage facings that met Perris’ HCO standards in Perris relative to comparison stores, stratified by (1) location within the checkout (considering product facings at all distances in the entire checkout) and (2) by store type (considering only facings within ≤6 ft of registers). Regarding location within the checkout, the baseline percentages of all facings that met HCO standards were highest at standalone displays (60%) and lowest at snaking sections (46%), with 50% at endcap and 51% at main checkouts. Baseline percentages of food and beverage facings that met standards (among food and beverage facings) were lowest at snaking sections (30%) and similar at other sections: endcaps (37%), main checkout (36%), and standalone displays (34%). Post-implementation, significant relative increases in the percentage of facings that met HCO standards were only observed at main checkout areas among all facings (32%; PR = 1.32; 95% CI [1.08,1.62]; *p = 0.007*), which increased from 51% to 65%, and among food and beverage facings (48%; PR = 1.48; 95% CI [1.09,2.00]; *p = 0.011*), which increased from 36% to 53%. No significant increases in meeting HCO standards were found for standalone displays, snaking sections, or endcaps (*ps* > 0.05).

### Changes in compliance by store type (≤6 ft of registers)

Regarding store type, baseline percentages of all facings that were compliant in Perris were lowest in independent grocery stores (45%) and highest in mass merchandisers (70%). Among food and beverage facings, baseline compliance was highest in mass merchandisers (56%) and chain supermarkets (38%), and lowest in dollar stores (24%) and independent grocery stores (30%). The two store types with the highest baseline percentages—mass merchandisers and chain supermarkets—were also the only store types with significant relative increases in post-implementation compliance in Perris relative to comparison cities (mass merchandisers: among all facings PR = 1.40; 95% CI [1.24,1.58]; *p < 0.001*; among food and beverage facings PR = 1.67; 95% CI [1.56,1.79]; *p < 0.001*; chain supermarkets: among all facings PR = 1.67; 95% CI [1.17,2.38]; *p = 0.005*; among food and beverage facings PR = 1.81; 95% CI [1.15,2.83]; *p = 0.010*). Post-implementation compliance was close to 100% for mass merchandisers (98% for all product facings and 96% for food and beverage facings). Post-implementation compliance in independent grocery stores was the lowest (45% for all facings and 30% for food and beverage facings). Store-level compliance rates for all 13 Perris stores are reported in S3 Table.

## Discussion

The observed store checkout environments’ healthfulness improved six months after implementation (one year after the original implementation date) of the HCO in Perris, CA. Relative to the comparison cities, Perris stores showed a 37% increase in the percentage of checkout facings ≤6 ft from the register that were HCO compliant, from 50% before implementation to 67% after. When considering the entire checkout area though, improvements were attenuated, and improvements >6 ft from the register were modest and not statistically significant. Consistent with this observation was our finding that only the main checkout area (and not the endcaps, standalone displays and snaking sections that tend to be further from the register) showed significant improvements in the percentage of facings meeting HCO standards. So, while Perris customers will be exposed to fewer noncompliant products, they may still encounter them on a typical checkout journey. When focusing only on food and beverage facings ≤6 ft from the register, the increase in compliance was greater: a 52% increase in compliance relative to the comparison cities, from 36% before implementation to 55% after. Among food and beverage facings, we observed an increased percentage that were compliant beverages (in particular, 100% fruit juice). When considering the entire checkout, improvements in the percentage of facings meeting HCO standards were found in the main checkout area. When we examined overall changes in compliance by store type, Perris’ mass merchandisers and chain supermarkets improved the most. In summary, the Perris HCO improved the healthfulness of store checkout environments, particularly within the policy’s targeted 6-ft zone, though about one-third of facings remained noncompliant post-implementation, suggesting that stronger policy design and enforcement could drive further change.

Our results are consistent with findings from previous studies that healthy checkout policies can improve store checkout environments [19,20]. As over one-third of adults reported buying a food or drink found at checkout during their last grocery trip, store checkouts represent an important setting for public health intervention [6]. The hypothesized theory of change is that such policies improve the retail food environment and, in turn, influence purchasing behavior and population diets [30–32]. With respect to the retail food environment, voluntary but meaningful healthy checkout policies in the UK have been associated with reduced exposure to less healthy foods and beverages at checkouts in cross-sectional studies [32,33]. In Berkeley, California—the first jurisdiction in the world to implement a mandatory healthy checkout policy—compliance among all product facings increased by 63% relative to comparison cities one year after implementation, and by 125% among food and beverage facings [19]. This corresponded to a 70% reduction in added sugar per serving for foods and beverages [20]. With respect to consumer behavior, evaluations of experimental interventions consistently show that such interventions reduced unhealthy purchases and increased purchases of healthier alternatives [16–18,34,35]. Real-world evidence points in the same direction. In the UK, voluntary healthy checkout policies led to 17% fewer purchases of small unhealthy items after supermarkets announced the changes [15]. England’s mandatory placement restriction, which prohibits displaying HFSS foods and beverages in prominent store locations such as checkouts and aisle endcaps, reduced sales of unhealthy items by an estimated 2 million items per day, which could have meaningful implications on population diets [9]. In Berkeley, California, implementation of the mandatory healthy checkout policy was associated with a 23% reduction in average soda sales [36].

Although HCOs have been shown to improve the food environment at checkout, the extent to which they are effective may in part depend on policy design [19,20,37]. At the time the Perris HCO was implemented, the only three mandatory nutrition policies globally targeting checkout areas—Perris’ HCO, Berkeley’s HCO, and England’s HFSS placement restrictions—differed in policy scope and nutrition standards [11–13].

In terms of policy scope, Perris’ HCO initially targeted the entire checkout area, but following concessions to trade association and industry pushback, amendments narrowed its scope to just within 6 ft of registers [25,38]. Consistent with the narrower policy scope, improvements were more pronounced ≤6 ft of registers in Perris. Products >6 ft showed minimal improvement, despite being part of the checkout. As only 57% of all checkout facings were located ≤6 ft of registers, targeting the entire checkout area, as in Berkeley, could help drive larger improvements. Policies like England’s, which target multiple prime locations, may further reduce potential loopholes (e.g., moving non-permissible products to aisle ends [i.e., endcap] or other prime store locations).

In terms of nutrition standards, while England’s policy uses a nutrient profiling model to classify products as HFSS, Berkeley and Perris set their own standards. Of these two, Perris has more complex but also more lenient HCO standards compared to Berkeley. For example, foods with the first ingredient being fruit, vegetable, nut, seed, or legume are deemed compliant regardless of their nutrient profile, whereas in Berkeley, these food groups also must satisfy nutrient standards. This may have resulted in Berkeley experiencing a more substantial increase in nonfood and nonbeverage facings at checkouts [19] than occurred in Perris—perhaps because stores in Perris could substitute with a wider range of foods or beverages under the more lenient standards or because they were overall less able or willing to comply.

How and when jurisdictions implemented and enforced the policy may also have driven differences in compliance. Post-implementation compliance ≤6 ft and the degree to which products in the entire checkout met their city’s HCO standards were both substantially lower in Perris compared to Berkeley (Perris: 37% increase ≤6 ft reaching 67% [33% noncompliance], and 31% increase in the entire checkout reaching 66%; Berkeley: 63% increase in overall compliance reaching 83% [17% noncompliance] [19]). Because implementation of the Perris HCO was delayed by six months, this evaluation measured outcomes at six months post-implementation in Perris, compared with 12 months post-implementation in Berkeley [12,38]. Lower compliance in Perris could in part be due to the shorter post-implementation timeline. Policy communication and enforcement may have also played a role. Although we do not have data on how the final HCO was implemented in Perris, in Berkeley, policy communication was conducted by a community organization that sent information packets and visited stores to provide technical support, while enforcement has been delayed due to capacity [25]. Evidence from England suggests that insufficient enforcement resources and policy complexity can hinder enforcement and thus limit store compliance [39,40].

These differences in policy design (and potentially implementation and enforcement) also appear to have influenced outcomes at the product category level. For example, the percentage of noncompliant SSBs did not change significantly in Perris, in contrast to the substantial reductions seen in Berkeley. The Perris HCO likely had limited impact on overall SSB availability because most beverages were on endcaps typically located beyond the 6 ft zone [1]. Reductions in the percentage of food and beverage facings that were noncompliant candy and other sweets were also smaller compared to Berkeley’s stores (e.g., candy: 79% reduction in Berkeley versus 32% nonsignificant reduction in Perris). Compliant fruits and vegetables [strict definition] also made up about 1% of food and beverage facings post-implementation, which may reflect standards that classify products by first ingredient and exempt categories like fruits and vegetables from nutrient limits. The smaller effects observed in Perris than in Berkeley across key noncompliant categories, combined with the low percentage of fruits and vegetables, suggest that the public health impact of Perris’ HCO may be more modest than policies with broader scope and stricter standards.

Even when policies are well-designed and enforced, retailers may respond in ways that undermine their intended effects. In Perris, the overall number of product facings decreased, which may in part be due to products being replaced with nonfood or nonbeverage items (e.g., toiletries, medicine) that tend to be physically larger than the product facings they replaced. In England, retailers responding to the policy reported relocating noncompliant products to unrestricted areas (e.g., in-aisle displays), replacing them with digital advertisements for restricted products, and increasing alcohol and nonfood items in restricted locations [39].

A strength of this study is the development and use of a large product-level dataset (>65,000 product facings) that included a near-census of products at sampled checkouts. The study is not without limitations. First, Perris HCO standards are complex, and although we relied on information and examples of compliant products provided by the City of Perris to determine compliance, some misclassification was possible. Second, while our Perris sample represents a census of all stores subject to the ordinance, some store types had very low sample sizes (e.g., one mass merchandiser), limiting broader generalizability of store type-specific findings to other contexts. Third, a small proportion (~5%) of product facings could not be assessed for compliance due to photographic obstructions, missing nutritional information, or out-of-stock products. Fourth, the analysis was limited to two time points and assumed parallel trends in the comparison cities, which cannot be formally tested without additional pre-policy data. However, comparison stores were matched to Perris stores by chain, and checkout environments in comparison cities changed very little over the study period, suggesting the improvements in Perris were driven by the policy rather than secular trends. Fifth, areas ≤6 ft of self-checkout registers but not considered part of the checkout area (e.g., shelves on the backside of a register inaccessible to customers in the checkout area) were not included in the analysis. Finally, estimating the 6-ft boundary from photos rather than in-person measurements may have introduced error.

Taken together, our findings show that Perris’ HCO improved the checkout food environment six months post-implementation, although the effect was smaller compared to Berkeley’s HCO one-year post-implementation. For jurisdictions considering similar policies, adopting Berkeley’s standards and applying them to the entire checkout area may be more effective. Future research should compare how stores interpreted and implemented this policy, how cities communicated HCO requirements to retailers and enforced the HCO, how the HCO affected product availability in the rest of the store, and what customers notice in the checkout area. Longer-term evaluations are also needed to assess how compliance changes over time, how customer purchases change in response, and whether these policies lead to improvements in population dietary intake and health outcomes.

## Supporting information

S1 Table Type and location of included stores in Perris, Moreno Valley, Rialto, and Riverside, CA. (DOCX)

S2 Table Sociodemographic characteristics for Perris and comparison cities. (DOCX)

S1 Checklist STROBE checklist for cross-sectional studies. The STROBE checklist is best used in conjunction with this article (freely available on the Web sites of PLoS Medicine at http://www.plosmedicine.org/, Annals of Internal Medicine at http://www.annals.org/, and Epidemiology at http://www.epidem.com/). Information on the STROBE Initiative is available at www.strobe-statement.org.
(DOC)

S1 File Methods for determining product distance from checkout registers. (PDF)

S1 Fig Compliance with Perris’ healthy checkout ordinance (HCO) standards among all facings and only food and beverage facings in Perris, CA. Abbreviations: comp, comparison cities; bev, beverages; PR, probability ratio (percentage change in Perris relative to comparison cities). **p < 0.01, *p < 0.05. Analyses included only facings within 6 ft of registers to assess compliance. Denominators for percentage compliant differ for percentage of all facings versus percentage of food and beverage facings. For adjusted estimates, marginal means were model-adjusted predicted percentages of facings that met HCO standards for each combination of treatment group (Perris vs. comparison cities) and time period (pre vs. post), averaged over store types in the sample. Estimates were derived from difference-in-differences Poisson regression models with robust standard errors clustered by store. *P* values are from two-sided Wald tests.
(TIF)

S2 Fig Percentage of all facings (left) and only food and beverage facings (right) in Perris, CA evaluated separately under Perris’ and Berkeley’s healthy checkout ordinance (HCO) standards. Abbreviations: comp, comparison cities; bev, beverages; PR, probability ratio (percentage change in Perris relative to comparison cities). **p < 0.01, *p < 0.05. Only included facings within 6 ft of registers to allow a like-for-like comparison of the HCO food and nutritional standards. The denominator for the all facings outcome was all product facings; the denominator for the food and beverage outcome was food and beverage facings. *P* values are from separate two-sided Wald tests using the Perris and Berkeley standards, with standard errors clustered by store.
(TIF)

S3 Fig Average number of product facings and facings that met healthy checkout ordinance (HCO) standards per store, before and six months after implementation of the policy in Perris and comparison cities, for all product facings (panel A) and facings within 6 ft (policy zone; panel B). **Abbreviations**: nonfood/nonbev, nonfood or nonbeverage; comp, comparison cities.
(TIF)

S3 Table Store-level compliance with Perris’ healthy checkout ordinance (HCO) standards within 6 ft of registers for each of the 13 Perris stores, six months after implementation. (DOCX)

## Abbreviations

DiD: difference-in-differences
HCO: healthy checkout ordinance
HFSS: high saturated fat, salt, or sugar
PR: probability ratio
SSB: sugar-sweetened beverage
