# Supplementary material for: The retail food environment following a mandatory healthy checkout policy: A natural experimental evaluation
Source: PLoS Med. 2026 Sep 16;23(9):e1004814. doi: 10.1371/journal.pmed.1004814 (PMC13581021; doi:10.1371/journal.pmed.1004814)
Supplement: Supplementary file 1 [file pmed.1004814.s004.pdf]

File S1. Methods for determining product distance from checkout registers

In Summation:

Referencing photographs collected during 2023 and 2024, items were designated into one of three categories (≤6, >6-≤9, >9 feet) based on their estimated distance from store registers. The center point for establishing radii was defined as where a customer and cashier interact to perform a transaction, such as a card reader, scanner, or touch screen. Zone boundaries were informed by the dimensions of flooring tiles, products, and physical structures using strategies such as point-perspective principles, digital to physical scaling, geometric mapping, and grid-based diagrams.

Extended:

Items were grouped into one of three categories based on their estimated distance from any checkout registers: ≤6, >6-≤9, >9 feet. The exact point of origin for measurement was interpreted as where a patron interacts with the register or cashier to perform a transaction, including those that were present but not assessed. The three zones extend in all directions to include the end and opposite sides of a counter or lane. Grouped self-checkout areas varied slightly, determined on a case-by-case basis for snaking sections. In reviewing photographs from a store's assessment, those from the original visit were prioritized unless they were deemed unreliable or if a store faced major structural changes. Retroactively determining the boundaries of each zone involved referencing the dimensions of a combination of three key elements: flooring tiles, products, and physical structures. Each store required a slightly unique method, thus each was noted and visually imposed on existing photos into a slide deck for future replicability.

Determining tile size in stores with uniform flooring allowed for tile counting with the opportunity to translate information into a grid-based model of checkout for confirming boundary edges. Estimation involved comparing dimensions of products and physical structures that closely aligned with tile edges in photos. Selecting an image with a direct view of products, preferably with a hard edge, or a standardized structure, such as commercial fridges or freezers, provided the most reliable points-of-reference. Application of point-perspective principles identifies references for tile counting when the register and items were distanced from the floor. Point-perspective drawing is a technique for illustrating depth based on horizon lines and vanishing points; reversing the process by imposing a series of parallel and adjacent lines on photographs pinpointed these approximate locations. **Figure 1** is an example of point-perspective application where the red, vertical line was the final approximation of register location.

Figure 1: Overlay of Perspective Lines on a Standard Checkout

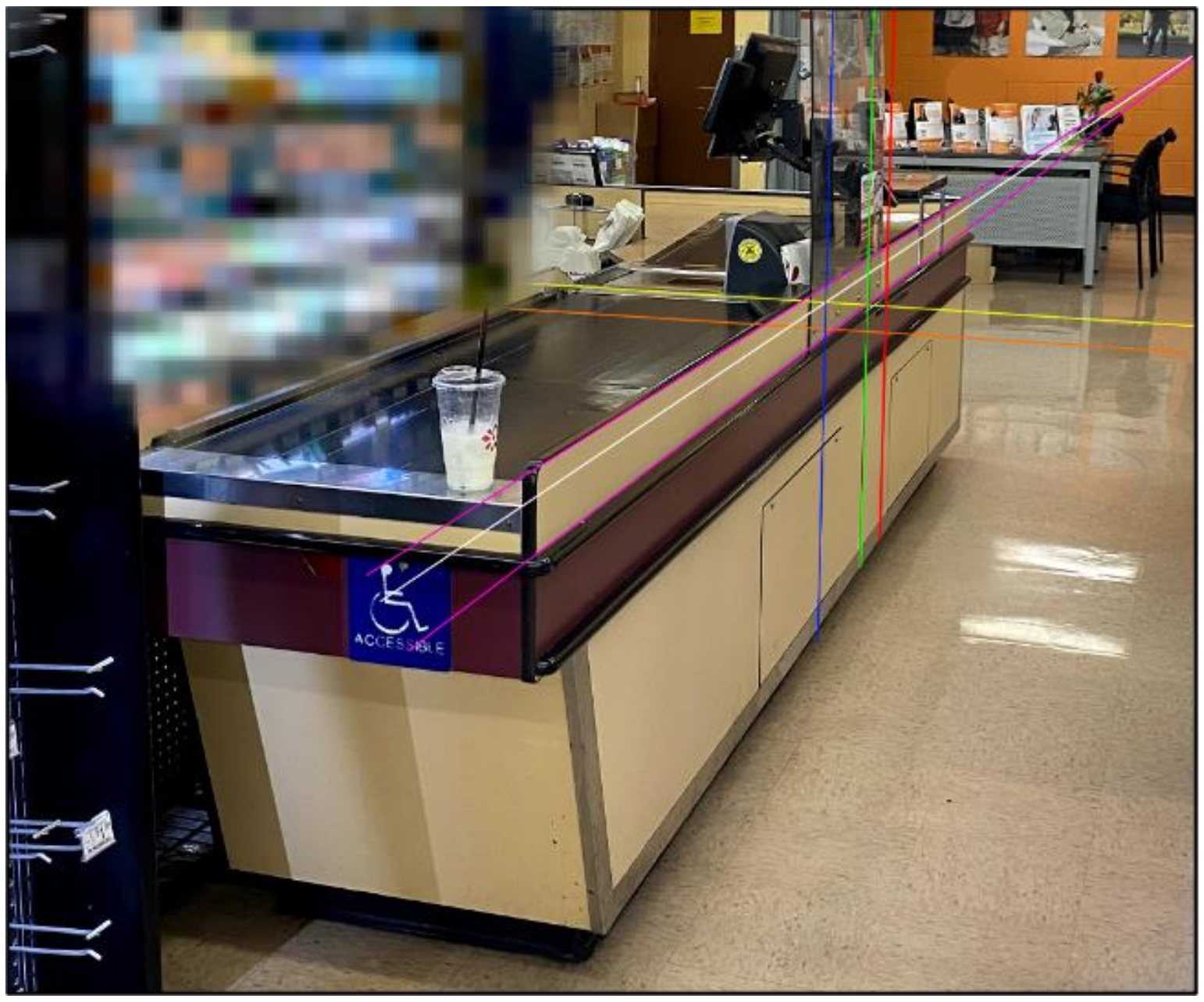

Zone boundaries in stores without tiles were informed by items found at checkout and structural reference points, such as pegboards or commercial fridges. Selecting a well-stocked shelf with repeated products, such as gum packages, provided a reliable base estimation of shelf length with consideration of stock spacing and physical framework. Dimensions were gauged via online retailer listings; however, inconsistencies across sellers were common and required cross-checking. This approach, independent of floor tiles, requires careful attention to spatial distances where no solid reference points are available, such as walkways or empty counters. Estimating the length and width of a counter or conveyor belt informed radial boundaries for geometric mapping (**Figure 2**) similar to, but less precise than, tile grid-based modeling (**Figure 3**). Both styles of mapping clarify zone borders for items distant or disconnected from the main checkout structure, such as standalones and snaking lines.

Figure 2: Geometric Mapping for Determining Standalone Distance

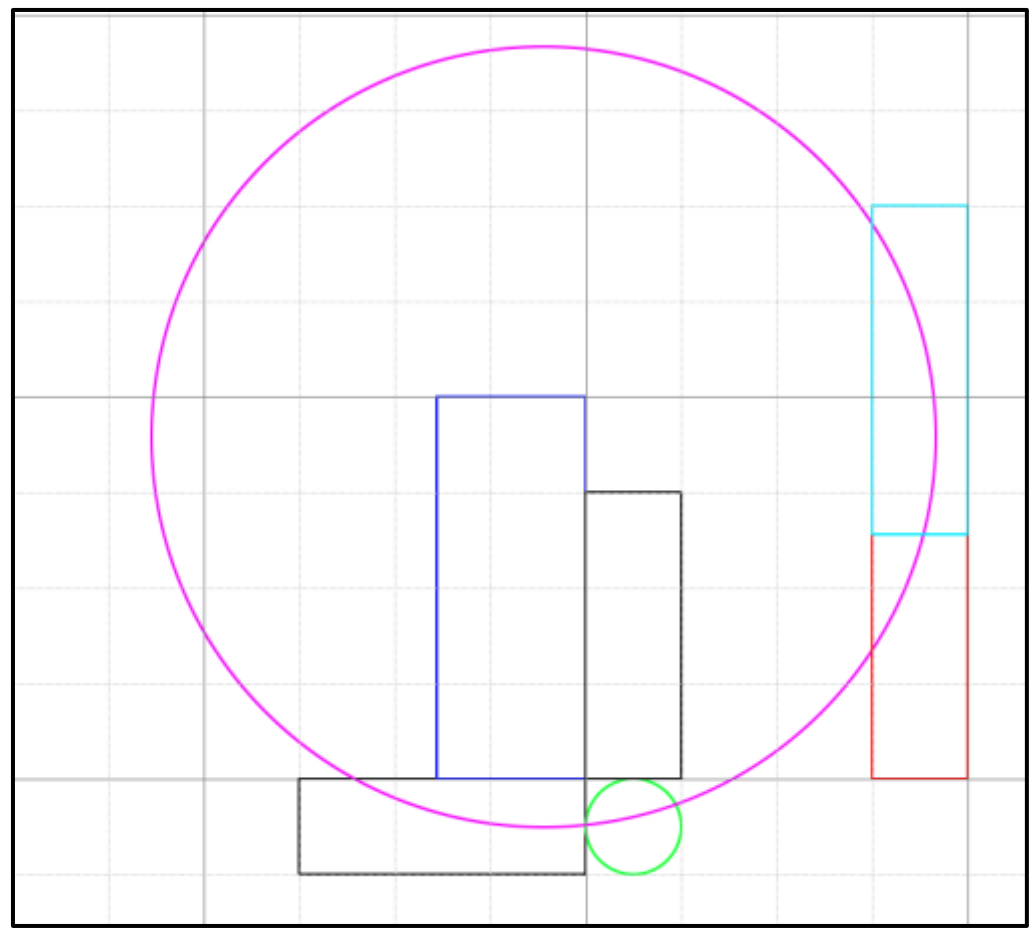

Figure 3: Grid-based Mapping for Determining Snaking Structure Distance

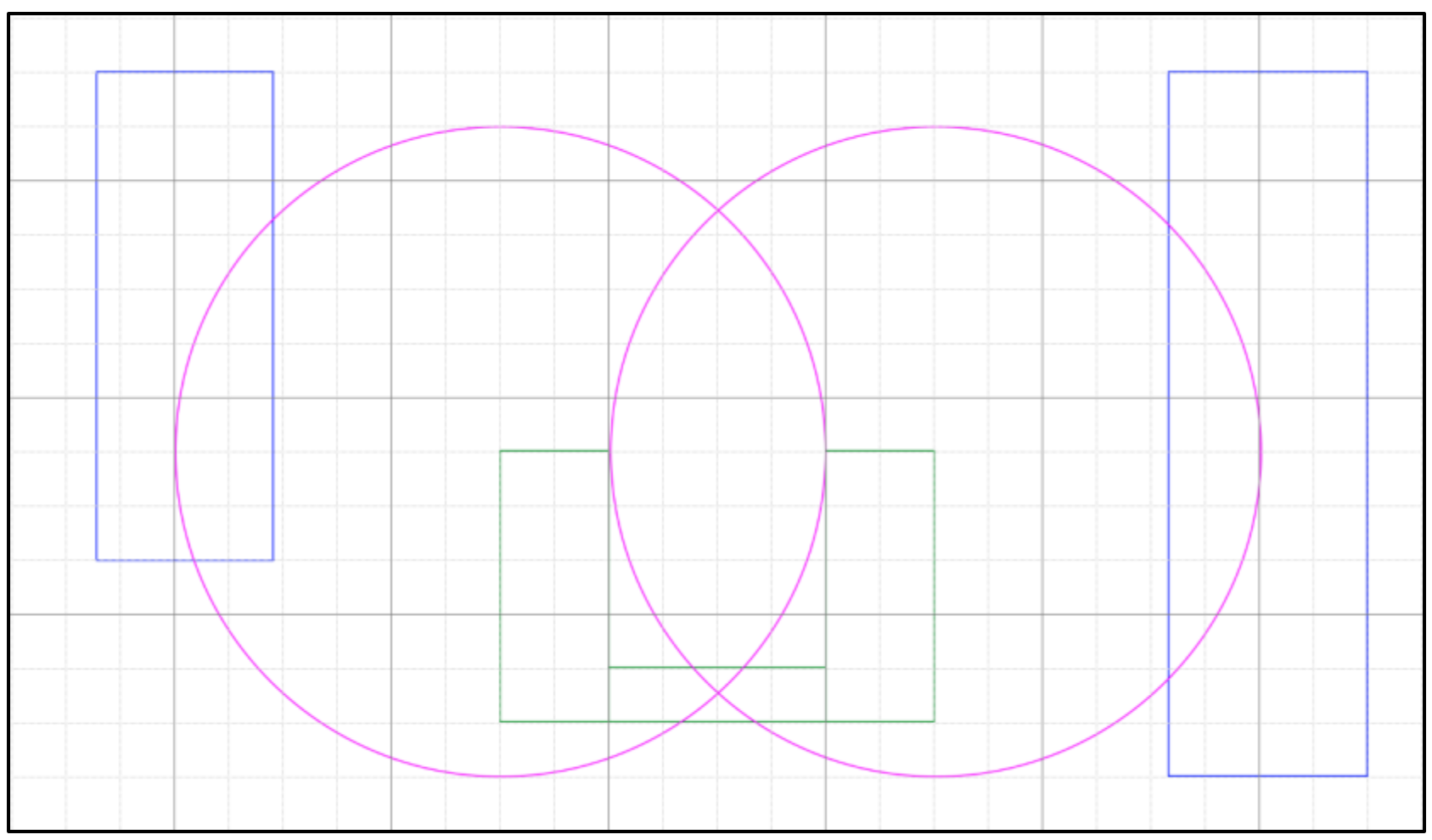

Digital to physical scaling was a final, infrequently utilized, approach similar to the tile method: setting the unit of scale as a physical ruler held to the computer monitor while maintaining zoom percentage and movement. This method was least favored due to difficulty in controlling external factors, such as screen movement or imprecise alignment. By measuring an item in the image with known dimensions, a ratio of digital to physical length could be established and applied to other sections of checkout. This approach requires fluidity in adapting the ratio in relation to dimensional positioning, requiring aid from the point-perspective method.

Limitations:

- Self-checkout and snaking structures required individualized determination of areas for inclusion, as some standalones were within the boundary of registers, but completely inaccessible to customers either in line or at the checkout. Other products, such as stacked drink boxes, were arranged displays for ushering customers, thus impossible to take from.
- Measuring distance by product dimensions requires additional buffer space due to variability in product sizing on online retailer listings, spacing between items, and structural variability, thus increasing ambiguity.
- Differences in photographer positioning and a lack of photographs directly facing checkout register areas warps perception of depth and angles, decreasing accuracy in pinpointing a reference line or point.
