# Supplementary material for: The retail food environment following a mandatory healthy checkout policy: A natural experimental evaluation
Source: PLoS Med. 2026 Sep 16;23(9):e1004814. doi: 10.1371/journal.pmed.1004814 (PMC13581021; doi:10.1371/journal.pmed.1004814)
Supplement: Supplementary file 2 [file pmed.1004814.s001.docx]

**S1 Table** Type and location of included stores in Perris, Moreno Valley, Rialto, and Riverside, California

| Store type | **Perris, *n (%)*** | **Moreno Valley, *n (%)*** | **Rialto, *n (%)*** | **Riverside, *n (%)*** | **All cities, *n (%)*** |
| --- | --- | --- | --- | --- | --- |
| Chain Supermarket | 6 (46.2) | 8 (50.0) | 5 (38.5) | 8 (36.4) | 27 (42.2) |
| Dollar Store | 2 (15.4) | 1 (6.2) | 3 (23.1) | 1 (4.5) | 7 (10.9) |
| Independent Grocery | 4 (30.8) | 6 (37.5) | 4 (30.8) | 11 (50.0) | 25 (39.1) |
| Mass Merchandiser | 1 (7.7) | 1 (6.2) | 1 (7.7) | 2 (9.1) | 5 (7.8) |
| **Total** | 13 | 16 | 13 | 22 | 64 |

Note: Stores from comparison cities were selected using stratified random sampling to match store type and chain in Perris when possible. When this was not possible (e.g., due there being an insufficient number of stores), additional stores within a type or chain were sampled from a different comparison city. When possible, extra stores within a store type were sampled from comparison cities to account for possibility of store closures or refusals between baseline and follow-up.
