# Supplementary material for: The retail food environment following a mandatory healthy checkout policy: A natural experimental evaluation
Source: PLoS Med. 2026 Sep 16;23(9):e1004814. doi: 10.1371/journal.pmed.1004814 (PMC13581021; doi:10.1371/journal.pmed.1004814)
Supplement: Supplementary file 3 [file pmed.1004814.s002.docx]

**S2 Table** Sociodemographic characteristics for Perris and comparison cities in California

|  | **Perris** |  | **Nearby Cities** | | |
| --- | --- | --- | --- | --- | --- |
| **Sociodemographics^a^** |  |  | **Moreno Valley** | **Rialto** | **Riverside** |
| Hispanic, % | 78.3% |  | 59.7% | 74.3% | 55.0% |
| Median Household Income, $ | $82,523 |  | $87,477 | $85,521 | $88,575 |
| Below federal poverty level, % | 11.9% |  | 11.8% | 12.6% | 11.3% |
| Bachelor’s degree or higher, % | 11.6% |  | 16.3% | 12.5% | 24.0% |
| Distance from Perris, mi | N/A |  | 14 mi | 26 mi | 19 mi |

^a^ Population estimates are from the U.S. Census Bureau, 2019–2023 American Community Survey 5-year estimates
