# Supplementary material for: The retail food environment following a mandatory healthy checkout policy: A natural experimental evaluation
Source: PLoS Med. 2026 Sep 16;23(9):e1004814. doi: 10.1371/journal.pmed.1004814 (PMC13581021; doi:10.1371/journal.pmed.1004814)
Supplement: Supplementary file 4 [file pmed.1004814.s008.docx]

**S3 Table.** Store-level compliance with Perris healthy checkout ordinance (HCO) standards within 6-ft of registers, six months after implementation

| **Store Type** | **Total Facings (≤6 ft)** | **Compliant Facings** | **Compliance (%)** |
| --- | --- | --- | --- |
| Chain Supermarket | 68 | 68 | 100.0 |
| Chain Supermarket | 306 | 299 | 97.7 |
| Chain Supermarket | 302 | 277 | 91.7 |
| Chain Supermarket | 175 | 157 | 89.7 |
| Chain Supermarket | 443 | 242 | 54.6 |
| Chain Supermarket | 491 | 259 | 52.7 |
| Dollar Store | 220 | 202 | 91.8 |
| Dollar Store | 223 | 157 | 70.4 |
| Independent Grocery | 97 | 90 | 92.8 |
| Independent Grocery | 157 | 81 | 51.6 |
| Independent Grocery | 558 | 226 | 40.5 |
| Independent Grocery | 313 | 114 | 36.4 |
| Mass Merchandiser | 242 | 237 | 97.9 |

**Note:** Compliance is defined as the percentage of all product facings within 6 ft of registers that met Perris HCO standards. All nonfood and nonbeverage facings are deemed compliant. Data are from wave 2 (June 2024), six months after policy implementation.
